# Group counselling for adherence support among young people failing first-line antiretroviral therapy in Zimbabwe

**DOI:** 10.4102/sajhivmed.v22i1.1292

**Published:** 2021-10-29

**Authors:** Bahati Kasimonje, Tinei Shamu, Tinashe Mudzviti, Ruedi Luethy

**Affiliations:** 1 Newlands Clinic, Harare, Zimbabwe; 2 Institute of Social and Preventive Medicine, University of Bern, Bern, Switzerland; 3 Graduate School of Health Sciences, University of Bern, Bern, Switzerland; 4 Department of Pharmacy and Pharmaceutical Sciences, University of Zimbabwe, Harare, Zimbabwe

**Keywords:** enhanced adherence counselling, adolescents, mental health, antiretroviral therapy, virological failure

## Abstract

**Background:**

Sub-optimal adherence to antiretroviral therapy (ART) is reportedly worse amongst young people living with HIV (YPLHIV). Group adherence counselling can be useful to improve adherence.

**Objectives:**

We evaluated an enhanced adherence counselling group intervention (EACGI) amongst YPLHIV failing a non-nucleoside reverse transcriptase (NNRTI)-based first-line ART regimen.

**Method:**

This was a retrospective cohort study using routinely collected data of YPLHIV failing NNRTI-based first-line ART. Patients with confirmed virological failure were referred for EACGI, a 12-week curriculum of weekly, 1.5-h sessions accommodating 8–15 people per group. It aimed to facilitate readiness to switch to second-line ART and improve adherence through a mental health intervention. Viral loads of HIV were measured pre-EACGI; at baseline; 3, 6 and 12 months post switch.

**Results:**

Fifty-seven patients aged 13–25 years were invited to EACGI and followed for up to 48 weeks. Thirty-three (58%) patients attended at least four sessions, whilst 24 (42%) attended none. Amongst those who attended none, two (8%) were transferred out, three (13%) were lost to follow-up and two (8%) had died by week 48 of follow-up, whilst all who attended were still in care. By week 48, amongst patients still in care, 29%, 44% and 67% of those who attended no sessions, 4–9 and 10–12 sessions, respectively, had viral loads of < 50 copies/mL.

**Conclusion:**

An EACGI is a promising intervention for YPLHIV failing ART prior to treatment switch, leading to improved adherence. This study’s findings support the need for further enquiry into rigorous, evidence-based multilevel adherence interventions that are acceptable and effective for YPLHIV.

## Introduction

Sub-optimal adherence to antiretroviral therapy (ART) amongst people living with HIV (PLHIV) is the leading cause of virological failure (VF).^1,2^ This is reported to be more pronounced in young people and to some extent ‘characteristic’ during this growth phase.^3,4,5,6^ Young people are a diverse group of the population and are defined in this study to be in the age range of 13–25 years. One of the major challenges that healthcare professionals face is helping young people adhere to ART despite its well-known benefits in improving the overall health and quality of life.^7,8^ Globally, HIV is a leading cause of death amongst young people, which is associated with inadequate adherence support and poor retention in care.^9,10,11^ Given the high risk of VF, disease progression, drug resistance, limited treatment options and the ultimate threat to survival, understanding and examining adherence strategies that delve beyond a traditional clinical approach is imperative.^12^ This is more so in countries in sub-Saharan Africa, where more than 80% of the world’s 2.1 million adolescents infected with HIV live.^4^

Part of young people’s HIV management and care involves treatment switching from a failing ART regimen to a different class of antiretrovirals when VF occurs, most often because of inconsistent adherence.^13,14^ The fundamental goal of regimen switching is to achieve and sustain virological suppression without compromising future treatment options.^1,14^ Although treatment switching is an established clinical practice, there are limited data on the virological status of young people following a treatment switch.^15^

Group adherence counselling is often utilised as an intervention to improve adherence in routine HIV care.^16^ Studies have shown mixed results in its effectiveness and were mainly conducted in adult populations.^11^ A randomised controlled trial in Thailand found a statistically significant difference in treatment outcomes in 15- to 24-year-olds for the intervention group,^17^ whilst in South Africa there was no difference in treatment outcomes for a group adherence strategy delivered to young people and their caregivers.^18^ Experiences and outcomes of group psychotherapy as an adherence support intervention in young people have not been widely documented. In this study, we report virological suppression outcomes amongst adolescents and young people who were invited to an Enhanced Adherence Counselling Group Intervention (EACGI) after failing a non-nucleoside reverse transcriptase (NNRTI) based first-line ART regimen.

## Methods

### Study design and setting

This was a retrospective cohort study using routinely collected data of adolescents and young PLHIV (13–25 years) failing NNRTI-based first–line ART at Newlands Clinic during the period 2015–2016. This study sought to evaluate an EACGI for young PLHIV on ART adherence. Newlands Clinic is an urban HIV treatment centre in Zimbabwe and a referral outpatient clinic providing comprehensive HIV care, which is supported by the Ruedi Luethy Foundation, a private voluntary organisation. The model of care is nurse-led, doctor supervised, with four nurses and two doctors dedicated to the care of over a 1000 adolescents and young people predominately from marginalised communities.^19^ Psychological support offered by a psychologist and peer counsellor is provided for patients struggling to cope with issues that affect their treatment success. The clinic had 1102 patients aged 13–25 years in care as of 31 December 2020.

Viral load measurements were conducted using the COBAS^®^ Ampliprep^®^/Taqman48^®^ platform and the Roche HIV1 version 2.0 assay. According to the clinical guidelines, virological treatment failure was defined as two consecutive viral load measurements > 200 copies/mL at least 3 months apart. Virological suppression was defined as achieving a viral load < 50 copies/mL. Patients with low-level viraemia (51 copies/mL – 200 copies/mL) were managed by their respective nurses and were not referred to EACGI. Once virological treatment failure on first-line ART was confirmed, patients were referred for the EACGI, which was part of routine care, aimed to facilitate readiness to switch treatment to second-line ART, and improve adherence through phenomenological, motivational interviewing and cognitive behavioural therapy principles. All patients who were referred for EACGI were included in the study. The EACGI was a 12-week curriculum of weekly, 1.5-h sessions that accommodated 8–15 people per group during the period 2015–2016. The intervention was developed locally in the clinic by the psychologist through adapting the past experiences of clinicians, peer counsellors and patients. These experiences were then used to design the intervention to best meet the needs of adolescents failing treatment. Participants were conveniently selected to a group based on their respective ages (13–17 years and 18–24 years). Participants were informed and invited to attend EACGI by their respective nurse and peer counsellor. Group sessions were facilitated by a resident psychologist and a peer counsellor in English, the local dialect (Shona) and a youth friendly language. The peer counsellor was trained under the Africaid’s Community Adolescent Treatment Support programme.^20^ Each session began with an icebreaker to help to establish rapport, and create a positive group atmosphere and cohesion. Participants were offered bus fare and refreshments after each session. All participants who missed the initial EACGI sessions were followed up telephonically or at their clinic visit by their peer counsellor to establish barriers to attendance. Reasons for missing visits for those who attended the EACGI were asked during the individual interviews or at group session recaps. An overview of the EACGI curriculum is displayed in Table 1. This approach is published elsewhere.^21^

**TABLE 1:** Overview of the enhanced adherence counselling group intervention curriculum.

Week	Activity	Purpose
1	Introduction Conceptualising HIV, ART and adherence: Thoughts that come to mind when one thinks of HIV Thoughts that come to mind when one thinks of ART Thoughts that come to mind when one thinks of adherence	To outline the reason for referral and purpose of EACGI. To establish group etiquette and consent. Gives insight into meanings attached to one’s status, patients’ understanding of health and what adherence is.
2	Adherence facts quiz	To assess participants’ knowledge, regarding adherence, medication and HIV.
3	Discussion on HIV treatment literacy: Adherence, suppression, resistance, treatment options and psychosocial factors	To help participants understand their treatment and provide insight into how their thoughts about self and treatment affect adherence and health.
4	Recap of the initial three sessions	To summarise and remind participants of previously learnt information and seek clarity or ask any further questions. To enquire from participants of any topics or questions they would want to be addressed during group sessions.
5	Individual interviews: Each participant had allocated time to discuss their clinical management in private	To discuss and facilitate patient understanding of their laboratory and clinical data (viral load, CD4 count, opportunistic infections and treatment options). To provide a forum for patients to discuss individual barriers and enablers of adherence. To empower participants to take ownership of their health management.
6	Motivational interviewing exercise: Readiness ruler stages of change	To encourage participants’ motivation, confidence and readiness to adhere to medication.
7	Narrative exercise: Reflect and write a narrative on their journey with HIV	This gave insight on how members were processing their HIV status and allowed patients to seek group support for any difficulties or share stories of resilience.
8	Case study exercises: Stigma Depression Disclosure Participants’ choice	To develop strategies for coping with various psychosocial determinants of adherence.
9	Peer story: Peer counsellor Peer video (Me, Myself & HIV YouTube)	Provide peer experiences and support.
10	Question and answer session on barriers to adherence: Based on common barriers to adherence Participant contribution	To develop problem-solving skills and discover collective solutions for barriers to good adherence.
11	Letter writing exercise: Dear future self: Letters given back to participants as a reminder of goals post EACGI	To provide a referral point for future hopes and goals and how ART may play a positive role in achieving them.
12	Exit discussion	Clarification of anything that remains unclear or needs reinforcement. Participant feedback.

ART, antiretroviral therapy; EACGI, enhanced adherence counselling group intervention.

After undergoing EACGI, the patients who achieved virological suppression continued their current regimen, whilst those who were unsuppressed were switched to a second-line protease inhibitor-based regimen. Viral loads of HIV were measured pre-EACGI at the end of the 12-week sessions (time of switching to second-line ART), and at 3, 6, 9 and 12 months post switch to assess virological outcomes.

All patient data were recorded in the clinic’s electronic medical record (EMR), which is only accessible to authorised personnel and is password protected.

### Statistical analysis

We used frequencies and proportions to describe patient distributions in categories. For the description of central tendency, we used medians and interquartile ranges for variables not normally distributed. We measured normality of continuous variables using the Shapiro–Wilk test. Attendance rates were analysed as no attendance, < 75% and at least 75% attendance. The initial nine EACGI curriculum sessions covered the fundamental aspects of treatment literacy, whilst the final sessions reinforced taught material. We used logarithm transformed viral load measurements before comparing using two-sample *t*-tests. We used Stata version 13.1 (College Station, Texas, United States) and DABEST package (version 0.3.0) in R studio (R version 4.0.4 [2021 Feb 15]) for statistical analysis.^22^

### Ethical considerations

This study was approved by the Newlands Clinic Research Unit and the Medical Research Council of Zimbabwe (MRCZ/E/205).

All participants aged 18 years and older provided written informed consent before participating in this study. Participants younger than 18 years provided written assent, and their parents or guardians provided written informed consent.

## Results

**FIGURE 1:**
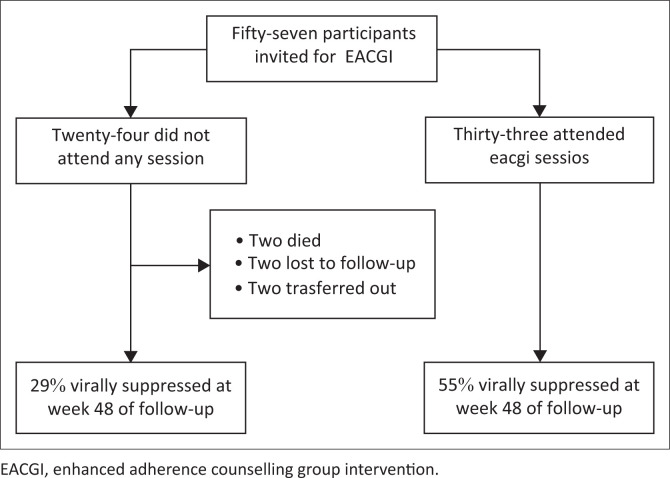
Flowchart showing participant enrolment and intervention outcomes.

Fifty-seven patients (*n* = 34, 60.0% female) aged 13–25 years were invited to the EACGI and followed up for 48 weeks. The median duration of first-line ART was 6 years (interquartile range [IQR]: 4–8) at the time of invitation to EACGI. Thirty-three (57.9%) patients attended at least four EACGI sessions, whilst the other 24 (42.1%) patients did not attend any session. Of the 33 patients who attended the EACGI, 25 (75.8%) were female patients, whilst 15 (62.5%) of those who did not attend were male patients. The main reasons for not attending the EACGI include a lack of interest and school or work commitments. Main reasons reported by patients for a low attendance rate to EACGI amongst those who attended were hopelessness, family dysfunction, perception of not having an illness, an aversion to a daily routine attached to stigma and prior experience of medication side effects. Amongst those who did not attend the EACGI, two (8%) were transferred out, three (13.0%) were lost to follow-up and two (8.0%) had died by week 48 of follow-up, whilst all who attended were still in care (Figure 1).

As shown in Table 2, the baseline demographic and treatment characteristics of the patients invited to attend the EACGI are listed.

**TABLE 2:** Demographic and treatment characteristics at the time of invitation to enhanced adherence counselling group intervention.

Characteristic	Enhanced adherence counselling group intervention (EACGI) attendance								Total (*N* = 57)			
	Did not attend any session (*n* = 24)				Attended at least one session (*n* = 33)							
	*n*	%	Median	IQR	*n*	%	Median	IQR	*n*	%	Median	IQR
**Gender**												
Male	15	62.5	-	-	8	24.2	-	-	23	40.4	-	-
Female	9	37.5	-	-	25	75.8	-	-	34	59.6	-	-
**Age (years)**			20	17–21			19	15–22			19	17–21
13–17	7	29.2	-	-	14	42.4	-	-	21	36.8	-	-
18–25	17	70.8	-	-	19	57.6	-	-	36	63.2	-	-
**Viral load (copies/mL)**	**-**	-	1635	210–33 118	-	-	24 547	2530–96 424	-	-	6228	466–60 652
**Attendance rate (sessions)**												
None	24	-	-	-	-	54.5	-	-	24	42.1	-	-
< 75%	-	-	-	-	18	45.5	-	-	18	31.6	-	-
≥ 75%	-	-	-	-	15	-	-	-	15	26.3	-	-

IQR, interquartile range.

Overall, there was a mean decline in the viral load of participants after EACGI before switching to second-line ART by 1.03 log copies/mL (standard error [SE]: 0.22, *p* < 0.001). Those who attended EACGI had the highest decline of 1.24 log copies/mL (SE: 0.29, *p* < 0.001) compared with 0.76 log copies/mL (SE: 0.34, *p* = 0.028) amongst those who did not attend.

**FIGURE 2:**
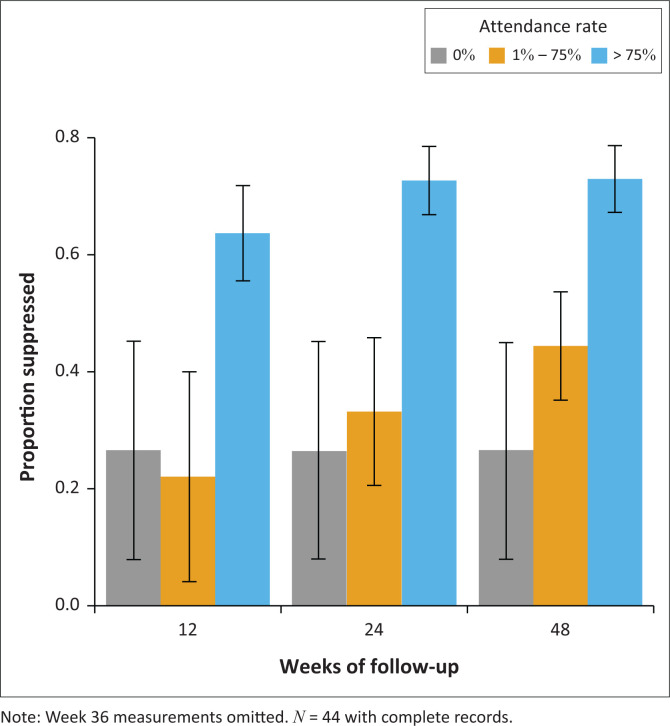
HIV viral suppression rates (< 50 copies/mL) according to the enhanced adherence counselling attendance rate.

**FIGURE 3:**
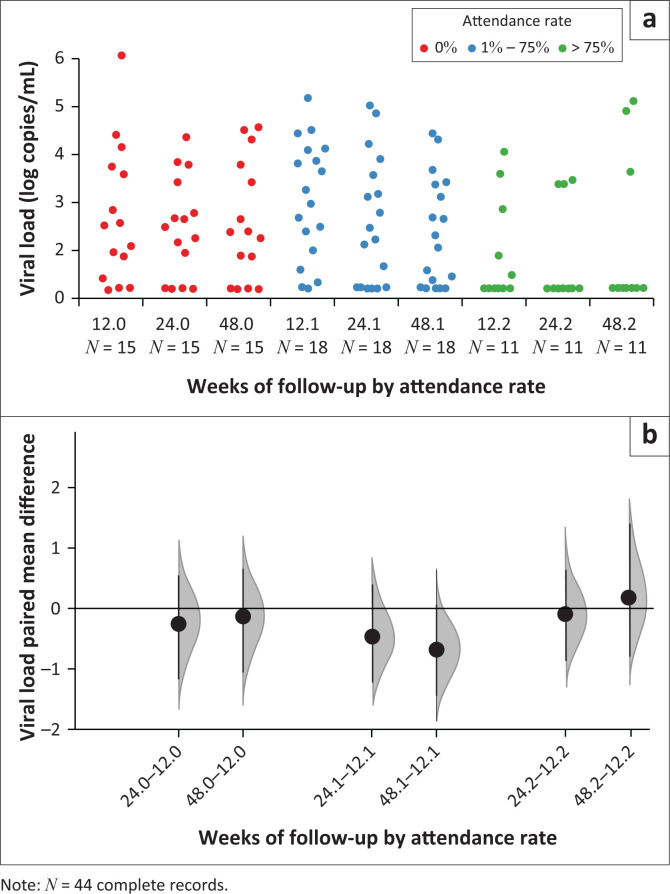
(a) Cumming estimation plot: raw data of log10 viral load measurements of patients at 12, 24 and 48 weeks of follow-up, (b) paired mean differences ± standard deviation in log10 viral load measurements and 95% confidence intervals (CI) at 24 and 48 weeks of follow-up where group 0 (denoted by follow-up 24.0, 48.0) are those with 0% attendance, group 1 (denoted by follow-up 24.1, 48.1) are those with attendance rates 1% – 75%, and group 2 (denoted by follow-up 24.2, 48.2) are those with attendance rates > 75%.

Amongst patients who attended > 75% of sessions, the viral suppression rate was 73% by week 12, but reduced to 64% at week 24 and to 67% at week 48 on second-line ART. Amongst those who attended four to nine sessions, the viral suppression rate increased from 17% to 44% by week 48. Virological suppression at week 48 was lowest amongst those who did not attend any of the sessions at 29% (Figure 2). Less than half of the participants had week 36 viral load measurements and, therefore, are not reported. Although viral loads were lowest amongst patients who attended the most sessions and highest amongst those who did not attend, a few patients who attended > 75% of sessions were failing second-line ART by week 48 (Figure 3).

## Discussion

We present the virological response of young people who were invited to an EACGI over one year of follow-up. The mental health adherence support intervention enabled improved adherence to ART and consequently positive virological outcomes on second line in young people who regularly attended group counselling sessions. Additionally, the difference in virological suppression was sustained over one year post intervention. All participants who attended the group intervention were retained in care during the 48 weeks of follow-up. It is especially noteworthy that this was achieved in young people who are ART experienced. Whilst the effect of an ART regimen switch alone may be sufficient to elicit positive virological outcomes because of the use of a more potent regimen, an EACGI could provide additional treatment support for adolescent patients.

Compared with other age groups in HIV care, adolescents show poorer adherence.^3,23^ A study in South Africa that investigated ART outcomes in young people attending public sector HIV clinics across two main provinces revealed that young people are more likely to have a detectable viral load 6 months post ART initiation compared with adults.^24^ The study also found that youth were more at risk of treatment failure when compared with adults.^24^ The findings of this study contribute to the literature on the intricacies of adolescent HIV care, the risk of adherence relapses and the likelihood of continued challenges with treatment outcomes in this population, which is characterised by social autonomy, increased risk-taking behaviour and a present bias, where immediate gratification, such as a ‘drug holiday’, is prized over the future long-term consequences of poor adherence.^15,25^

We postulate that offering more than the traditional biomedical education on the importance of adherence and including mental health in the intervention were significant mediating factors in contributing to adherence behaviour change and observed virological outcomes. Group sessions in the current study not only facilitated improved treatment literacy but also actively encouraged young people to take ownership and participate in their healthcare. Sessions also provided a space where mental health drivers of poor adherence were recognised, and participants were equipped with psychosocial skills to develop coping strategies. Peer support was also available throughout the intervention. Furthermore, the Newlands Clinic model is founded on comprehensive care consisting of a mental and social health service, which provides a context, in which young people experiencing difficulties with adherence can benefit from constant support to maintain adherence behaviour change. This aligns with the theoretical framework of offering youth friendly services, motivational interviewing, and cognitive behavioural therapy to change behaviour.^26,27,28^ These findings are consistent with the Thai study that showed the potential of an empowerment programme for youth in improving adherence to ART.^17^

The study results showed that young people who never attended the EACGI showed the lowest rates of suppression on the switched regimen. This finding highlights how, although essential, in the context of treatment failure, a regimen switch may not yield intended suppression results and may potentially increase the risk of drug resistance if adherence difficulties are not addressed. Unsuppressed viraemia in young people on second-line treatment may translate to potential public health consequences, as young people have the highest HIV incident and mortality rates, and the transmission of resistant strains leaves the newly infected with limited treatment options.^9,29^ This has healthcare costs in low-resource settings as subsequent treatment lines are significantly more expensive.

As already highlighted, group attendance was associated with viral response. Not all adolescents chose or were able to regularly attend sessions. This is reflective of young people’s realities where competing factors, such as seeking autonomy, an active social life, and educational and financial responsibilities, contend with HIV care engagement.^23,30^ Economic considerations in HIV support interventions have previously been described as pivotal for successful HIV management, given the prioritisation of income-generating opportunities that may interfere with attending facility-based interventions.^30,31^ In their study, they suggest that young people living with HIV (YPLHIV) are mainly from low-resourced communities such as those in the current study and, therefore, it is plausible that income-generating opportunities may interfere with attending facility-based interventions. These findings highlight the importance of offering differentiated adherence interventions. This phenomenon may affect male patients more as observed in this study where fewer male patients attended the EACGI regularly. Group counselling interventions may not always achieve similar results in young men living with HIV, highlighting the need for further research in this area.

There are some differentiated treatment strategies that have been developed to try and improve HIV treatment outcomes in adolescents and young people. Teen clubs are one such model that have achieved mixed outcomes in different settings. These clubs have been rolled out and evaluated in a few treatment programmes around Africa.^32^ Adolescents receiving care and enrolled in teen clubs in Windhoek, Namibia, did not show improved treatment outcomes compared, with the standard of care.^33^ Whilst there was marginal improvement in adherence (adjusted odds ratio [aOR]: 1.48, 95% CI: 1.16–1.90, *p* < 0.01) to ART in a Malawian study utilising teen clubs.^32^ The introduction of an EACGI, which has shown positive results in the Zimbabwean setting, could very well complement the differentiated models being rolled out for young people requiring adherence support.

The strength of this work was that adherence behaviour change was measured by viral load, an objective clinical marker of adherence and clinical outcomes. The strength of this study’s results also lies in the ability to measure viral progression longitudinally in young people after group counselling intervention, which is difficult to conduct in low- and middle-income countries. One of the limitations with this approach is that the observed viral suppression after switching to second-line ART could be as a result of switching to a more potent treatment regimen.

Biomarkers are not the only important factors in making clinical decisions in routine HIV management, especially in key populations.^34^ This study’s findings support these recommendations and provide a possible preliminary framework on support for young people being prepared for a treatment switch post treatment failure.

This study was conducted using routinely collected data where patients were not randomised into groups. Willingness to attend group sessions may have affected the rate of attendance and virological outcomes. The EACGI referrals were initiated by clinicians’ judgement and could have introduced a selection bias. However, this is reflective of typical HIV primary care. This study focused on individual-related factors of adherence; yet, adherence is complex with multiple interacting adherence risk factors.^31^ The EACGI can be included in HIV treatment programmes with the inclusion of trained personnel.

## Conclusion

Adherence support interventions, which include a mental health framework such as EACGI, in preparing young adult patients for second-line treatment seems to be a promising tool, increasing the likelihood of improved adherence and treatment outcomes. All participants who attended EACGI were retained in care. This study’s findings support the need for further enquiry into rigorous, evidence-based multilevel adherence interventions that are acceptable and effective for diverse young PLWHIV.
